# Hyperadrenergic Orthostatic Hypotension With Pure Peripheral Sympathetic Denervation Associated With Sjogren’s Syndrome

**DOI:** 10.7759/cureus.17805

**Published:** 2021-09-07

**Authors:** Junichiro Takahashi, Tadashi Umehara, Hidetaka Mitsumura, Hidetomo Murakami, Yasuyuki Iguchi

**Affiliations:** 1 Department of Neurology, Jikei University School of Medicine, Tokyo, JPN

**Keywords:** syncope, hyperadrenergic orthostatic hypotension, autonomic nerve dysfunction, head up tilt test, sjogren's syndrome, auto-immune neuropathy, postganglionic sympathetic denervation

## Abstract

Hyperadrenergic orthostatic hypertension (hyper OH), defined as OH with elevated levels of upright plasma norepinephrine (NE), is assumed to be a clinical expression of peripheral sympathetic denervation (PSD). Primary Sjogren’s syndrome (pSS) is an autoimmune disease that not only affects the exocrine glands but also develops autonomic neuropathy with PSD. We present a hyper OH with PSD possibly associated with pSS and successfully treated with intravenous immunoglobulin (IVIg). The case was a 60-year-old man who developed recurrent syncope on rapid standing from a sitting position. Head-up tilt test and NE infusion test showed hyper OH with PSD. This case report raises the possible associations between hyper OH and autonomic neuropathy as pSS developed.

## Introduction

Orthostatic hypotension (OH) is defined as systolic blood pressure (SBP) decreases of at least 20 mmHg or diastolic blood pressure (DBP) of at least 10 mmHg within three minutes of standing [1].

Reflecting on impaired sympathetic nervous system (SNS) tone, OH typically showed low levels of upright plasma norepinephrine (NE).

Dr. David Streeten described a small group of patients with mild OH and normal or elevated levels of upright plasma NE levels, and first coined the term “hyperadrenergic orthostatic hypotension” (hyper OH). They showed patients with an elevated plasma NE level > 600 pg/mL exhibited signs of greater responsiveness in the lower versus upper extremity to NE infusion, indicating SNS denervation preferentially in the lower limbs [2].

After that, Dr. Mar PL and his colleagues reported a subset of hyper OH. They compared hyper OH and non-hyper OH groups and showed hyper OH maybe the earlier form of typical OH according to the fact that the hyper OH group showed less severe orthostatic intolerance and shorter duration of background diseases such as Parkinson’s disease (PD) and diabetes mellitus (DM) than non-hyper OH group. In addition to the fact of SNS denervation preferentially occurred in the lower limbs showed by Dr. David Streeten, they concluded hyper OH is a clinical expression of partial autonomic neuropathy of the peripheral sympathetic nerves innervating the lower extremities, as well as certain types of neuropathy such as diabetic neuropathy showed length-dependent polyneuropathy in its earlier stage. When the lower extremities have been affected but the upper extremities not yet, the excessive amounts of NE may be produced with compensation by the sympathetic nerves innervating upper extremities via the baroreflex [3,4].

Thus, hyper OH may be an earlier form of typical OH, but the relationship between intrinsic diseases and hyper OH remains unclear.

Primary Sjogren’s syndrome (pSS) is an autoimmune disease that affects the exocrine glands, leading to the development of keratoconjunctivitis sicca and xerostomia. Extra glandular manifestations, including neuropathy, are also well known [5].

Some reports that autonomic neuropathy associated with pSS showed super-sensitivity to NE on the NE infusion test, indicating peripheral SNS denervation [6].

Thus, pSS could develop hyper OH as partial autonomic neuropathy of the peripheral sympathetic nerves, but few reported the relationship between the two diseases.

We reported a case of hyper OH due to peripheral SNS denervation possibly associated with pSS, which was suggested by the NE infusion test and other autonomic tests.

## Case presentation

A 60-year-old man developed recurrent syncope on rapid standing from a sitting position. The syncopal events were brief, and his consciousness recovered within a few seconds. However, the frequency of the syncope gradually increased over a two-year period, and it could occur several times a day. He presented to our hospital for evaluation. He had no prior history of cardiac arrhythmias, DM, pheochromocytoma, radiation or surgery of neck surroundings, or supine hypertension. At the age of 56 years, he developed bilateral abducens nerve paralysis associated with pSS. The diagnosis was made according to the ACR-EULAR criteria (2016) [7], including the symptoms of sicca and the presence of anti-SS-A/Ro antibody. Since then, he had been treated with oral methylprednisolone. However, his medication included no antihypertensive or psychotropic drugs. His family history was unremarkable. His general physical examination was normal. The detailed neurological examination showed autonomic dysfunction that manifested as repetitive postural intolerance. Compared to it, other manifestations of autonomic dysfunction, such as constipation, urination, erectile dysfunction, or dyshidrosis, were much milder. Motor, sensory, coordination and cognitive functions were normal.

The laboratory findings are summarized in Table 1. The serum anti-SS-A/Ro antibody level was high, at 135 U/mL, but levels of Vitamin B12 and HbA1c were normal. Both the α3 and β4 subunits of serum ganglionic acetylcholine receptor (gAChR) antibody, which are observed in autoimmune autonomic gangliopathy (AAG), were negative. The radiological findings were all normal; there was no atrophy or abnormal lesions on brain MRI, normal uptake in the whole striatum with a normal specific binding ratio on ^123^I-FP-CIT single-photon emission computerized tomography (SPECT) imaging, and normal cardiac uptake on cardiac ^123^I-meta-iodobenzylguanidine (^123^I-MIBG) scintigraphy. To examine the state of OH, the 10-min head-up tilt (HUT) test to 60° was performed with an evaluation of supine and upright NE and vasopressin concentrations in the morning following an overnight fast. The plasma was drawn with an indwelling catheter instead of a needle not to provoke a sympathetic surge from pain or anxiety with the supine blood draw. Five minutes after tilt-up, his consciousness level deteriorated, and the table was returned to the supine position. The test demonstrated a supine blood pressure (BP) of 116/72 mmHg and a heart rate (HR) of 55 bpm at baseline. After tilting up for five minutes, the patient’s BP dropped to 66/38 mmHg, with an increased HR of 88 bpm, showing exaggerated postural hypotension with a BP decrease of Δ50/34 mmHg and a compensatory HR surge of Δ33 bpm.

**Table 1:** Laboratory findings of the patient NGSP; National Glycohemoglobin Standardization Program, gAChR; ganglionic acetylcholine receptor, 123I-FP-CIT SPECT; N-(3-fluoropropyl)-2β-carbomethoxy-3β-(4-[123I] iodophenyl) nortropane single-photon emission computed tomography, 123I-MIBG scintigraphy; [123I] meta-iodobenzylguanidine myocardial scintigraphy, NE; Norepinephrine

Blood tests									
Anti SS-A/Ro antibody (U/mL)	135 (>7.0)								
Anti SS-B/La antibody (U/mL)	2 (>7.0)								
Vitamin B12 (pg/mL)	606 (233-914)								
HbA1c (NGSP) (%)	6.0 (4.6-6.2)								
gAChR antibody (nmol/L)	α3 subset: negative								
	β4 subset: negative								
Radiological tests									
^123^I-FP-CIT SPECT	Right: 5.78	Light: 6.57			Average: 6.17				
Cardiac ^123^I-MIBG scintigraphy	Early: 2.28 (2.2-4.0)	Delay: 1.87 (2.2-4.0)			Washout: 51.8 (<25)				
Norepinephrine infusion test									
	0 min	1 min			2 min			3 min	
Systolic blood pressure (mmHg)	126	151			155			166	
Diastolic blood pressure (mmHg)	64	72			79			72	
Heart rate (bmp)	47	47			48			43	

It was worth noting that NE was high in both supine and standing positions (844 pg/mL and 886 pg/mL, respectively) with a poor increase of ΔNE of 42 pg/mL from supine rest to standing.

Furthermore, The NE infusion test was developed to evaluate the failure of vasoconstriction [8].

After resting for five minutes in a supine position, NE was administered intravenously at 3 µg/min for three minutes as the usually used concentration to detect super-sensitivity to NE associated with denervation, and the cardiovascular responses including changes in systolic BP, diastolic BP, and HR were assessed (Table 1). The test demonstrated a supine BP of 126/64 mmHg and HR of 47 bpm at baseline. After infusion, his BP increased to 166/72 mmHg, which indicated super-sensitivity to NE, with an excessive increase compared to the normal range of increase in systolic BP of 11±6.1 mmHg and diastolic BP of 4.7±2.3 mmHg [9].

## Discussion

The laboratory findings showed a high titer of the serum anti-SS-A/Ro antibody level, but there were no findings expressing autonomic failure including Vitamin B12, HbA1c, and serum gAChR antibody.

His neurological findings are only OH, no Parkinsonism, and normal radiological findings of 123I-FP-CIT SPECT imaging and 123I-MIBG scintigraphy indicated a low likelihood of an initial clinical presentation of synucleinopathies such as Lewy body diseases or multiple system atrophy.

On HUT, the ΔHR/Δsystolic BP ratio was 0.66. The changing pattern of BP and HR after tilt-up seemed to be that of non-neurological OH, as defined by a BP drop > 20/10 mmHg with a significant increase in the ΔHR/Δsystolic BP ratio of >0.5 [10], but without evidence of hypovolemia.

Considering a refractory status to increased fluid and salt intake and the adequate increase of HR after tilt-up, it appears that dysfunction of vasoconstriction caused excessive gravitational pooling of blood in the veins of the lower limbs. The blood pooling led to the reduction of venous return, and the consequent decrease in BP induced a compensatory increase in HR, as observed in patients with hypovolemia [11,12].

He showed hyperadrenergic OH with a high concentration of NE as defined > 600 pg/mL on standing, which was inconsistent with the features of pure autonomic failure (PAF). In addition, the poor elevation of NE from supine rest to standing was observed. Decreased increase of NE and normal increased of ADH after tilting up indicated the peripheral SNS denervation. The denervation would produce concurrent decreases in both release and reuptake. Peripheral release of NE from SNS simply leads to decreased plasma NE levels. On the other hand, decreased reuptake of interval between nervous-receptor on vessel muscle led to increased plasma NE levels. Thus, plasma NE values were identified by the balance of how severely disrupted release and reuptake of NE. In hyper OH, continuously compensating produce of NE by upper extremities to maintain BP with dysfunction of lower SNS lead to the imbalance of disrupted release and reuptake. 

Also, the NE infusion test revealed super-sensitivity to NE. As the sensitivity of the vessels to NE increases with postganglionic sympathetic denervation, exogenous NE provides a greater increase in BP [13].

 The normal cardiac ^123^I-MIBG scan and compensatory increased HR on standing would suggest against post-ganglionic cardiac SNS denervation, but peripheral SNS denervation as lower extremities dominantly, indicating long SNS denervation innervating lower extremity was significantly disrupted but not affected short cardiac NE neurons on this condition of neuropathy.

These findings indicated that he showed purely vascular sympathetic neuropathy and preserved cardiac sympathetic nerve.

After underlying causes of dysautonomia such as AAG, paraneoplastic neuronal syndromes, and amyloidosis were carefully excluded, the immunological association of pSS with postganglionic sympathetic denervation was considered the pathophysiology of OH in this case. Finally, high-dose intravenous immunoglobulin (IVIg) therapy was tried for five consecutive days (400 mg/kg/day) to target the autonomic nerve neuropathy associated with pSS. The IVIg was tried on one course. At three weeks after IVIg, he showed no syncope and was discharged to his home. During the outpatient follow-up one month later after treatment, he still showed no symptoms on standing, indicating the hypothesis that his OH had been mediated by an autoimmune disturbance due to pSS.

## Conclusions

To the best of our knowledge, this is the first case of hyperadrenergic OH with postganglionic sympathetic denervation associated with pSS showing a good response to IVIg. The findings also reinforce the importance of assessing pSS patients for the presence of OH, since it can be successfully treated.
